# An Improved Protocol for Efficient Engraftment in NOD/LTSZ-SCIDIL-2Rγ^NULL^ Mice Allows HIV Replication and Development of Anti-HIV Immune Responses

**DOI:** 10.1371/journal.pone.0038491

**Published:** 2012-06-04

**Authors:** Maneesh Singh, Pratibha Singh, Gilles Gaudray, Lucia Musumeci, Caroline Thielen, Dolores Vaira, Claire Vandergeeten, Laurence Delacroix, Ellen Van Gulck, Guido Vanham, Laurence de Leval, Souad Rahmouni, Michel Moutschen

**Affiliations:** 1 Immunology and infectious diseases unit GIGA-I3, University of Liege, Liege, Belgium; 2 Experimental pathology unit GIGA-Cancer, University of Liege, Liege, Belgium; 3 Liege AIDS reference center, University of Liege, Liege, Belgium; 4 Virology Unit Institute of Tropical Medicine, University of Antwerpen, Antwerpen, Antwerpen, Belgium; South Texas Veterans Health Care System and University Health Science Center San Antonio, United States of America

## Abstract

Cord blood hematopoietic progenitor cells (CB-HPCs) transplanted immunodeficient NOD/LtsZ-scidIL2Rγ^null^ (NSG) and NOD/SCID/IL2Rγ^null^ (NOG) mice need efficient human cell engraftment for long-term HIV-1 replication studies. Total body irradiation (TBI) is a classical myeloablation regimen used to improve engraftment levels of human cells in these humanized mice. Some recent reports suggest the use of busulfan as a myeloablation regimen to transplant HPCs in neonatal and adult NSG mice. In the present study, we further ameliorated the busulfan myeloablation regimen with fresh CB-CD34+cell transplantation in 3–4 week old NSG mice. In this CB-CD34+transplanted NSG mice engraftment efficiency of human CD45+cell is over 90% in peripheral blood. Optimal engraftment promoted early and increased CD3+T cell levels, with better lymphoid tissue development and prolonged human cell chimerism over 300 days. These humanized NSG mice have shown long-lasting viremia after HIV-1JRCSF and HIV-1Bal inoculation through intravenous and rectal routes. We also saw a gradual decline of the CD4+T cell count, widespread immune activation, up-regulation of inflammation marker and microbial translocation after HIV-1 infection. Humanized NSG mice reconstituted according to our new protocol produced, moderate cellular and humoral immune responses to HIV-1 postinfection. We believe that NSG mice reconstituted according to our easy to use protocol will provide a better in vivo model for HIV-1 replication and anti-HIV-1 therapy trials.

## Introduction

In recent years, efforts have been made to reconstitute a functional human immune system in murine models [1], [2]. Multilineage differentiation and self-renewal capacity of CD34^+^ cells have been explored to reconstitute various kind of immunodeficient mice [3], [4], [5], [6], [7]. Third generation, NOD-Rag1nullIL2Rγ^null^, NOD/LtSz-scid/IL2Rγ^null^ (NSG) and NOD/SCID/IL2Rγ^null^ (NOG) mice lack common interleukin-2 receptor gamma chain (IL2Rγ). IL2Rγ chain deficiency inhibits natural killer cell differentiation and causes defects in innate immunity [8]. When transplanted with human HPCs after low-dose TBI, these strains of mice display higher levels of engraftment compared to what has been previously obtained with other immunodeficient mouse stocks [5], [7], [9], [10]. Satisfactory levels of human cell engraftment have usually been achieved after TBI conditioning and CD34^+^ cell transplantation in newborn or 8–9 week old mice [2], [3], [4], [5], [6], [7]. These humanized NSG and NOG mouse models have allowed sufficient levels of human cell chimerism and are suitable for HIV-1 infection studies [5], [7], [11].

Nevertheless, there is still room for improvingthe differentiation of lymphoid tissues in the reconstituted mice and reaching T cell counts sufficient to sustain long-term HIV replication. It is also of paramount importance to reproduce in such models the different types of non-specific and specific immune responses associated with HIV infection and influencing its prognosis (*i.e.* immune activation, immune senescence, immune exhaustion and specific anti-HIV responses).

It has been shown that myelosuppression generated by busulfan (1,4-Butanedioldimethanesulfonate) improves CD45^+^ cell engraftment in humanized NSG mice. An initial protocol used busulfan conditioning at 50 mg/kg and transplantation of 2×10^6^ CB-HPCs along with a cytokine cocktail for humanization of NSG mice [12]. However, in another study, busulfan at 40 mg/kg was lethal for NSG mice [13]. Neonatal NSG mice have also been pretreated with 15 mg/kg busulfan and transplanted following various protocols involving intrahepatic or facial vein injection of CD34^+^ cells [14], [15]. However, such technical procedures performed in neonatal mice remain delicate and require expertise.

In the present study, we further optimized the busulfan conditioning protocol by transplanting fresh CB-HPCs through tail vein injection in 3–4 week old NSG mice after 50 mg/kg busulfan treatment. This protocol allowed to improve human cell engraftment and to reach T cell levels that can support intense and prolonged HIV replication in NSG mice. It also permitted differentiation of human monocytes and dendritic cells (DCs) and improved the development of lymphoid structures such as lymph nodes and thymus. Overall, the survival of engrafted mice was lengthened. Interestingly, infected mice developed specific humoral and cell-mediated immune responses as well as signs of non-specific immune activation and senescence. This improved protocol provides an easy and suitable model for the study of HIV pathogenesis and the evaluation of new therapeutic approaches.

## Materials and Methods

### Mice

NOD/LtSz-scid/IL2Rγ^null^ (NSG) mice were purchased from Jackson Laboratory (Bar Harbor, Maine, USA). Mice were bred and kept in a specific pathogen-free animal facility of the GIGA-Research of University of Liège (Liège, Belgium). Mice were maintained in micro-isolator cages and fed with autoclaved food and water. The females as well as male mice were used in all the experiments. Animal handling was in agreement with national legislation and institutional guidelines. University of Liege ethical committee has approved the use of mice, ethical application approval number-670.

### Pretransplantation Conditioning and Transplantation of Human Cord Blood-Derived Hematopoietic Stem Cells in NSG Mice

Busulfan (Sigma Aldrich, Munich, Germany) was dissolved in DMSO and diluted with RPMI-1640. Busulfan 20 mg/kg or 30 mg/kg was administered by a single intraperitoneal injection. For higher doses (*i.e.* 50–60 mg/kg), the administration was split in two i.p. injections with a 12-hour delay. Human cord blood (CB) was provided by the cord blood bank of the University Hospital, Liege, Belgium. CB mononuclear cells were separated by Ficoll-Hypaque density gradient. CD34^+^ cells were positively selected by magnetic separation using a direct CD34^+^ cell isolation kit (Stem Cells Technologies, Grenoble, France) according to the manufacturer’s instruction. Sorted cells always had purity over 95%, when checked by flow cytometry. Cells were either immediately used for the transplantation or frozen in liquid nitrogen until further use. We transplanted 1 to 2×10^5^ frozen or 2×10^5^ fresh CD34^+^ cells by i.v. tail injection.

### Flow Cytometry

Peripheral blood was taken from the tail vein at different times after transplantation of CD34^+^ cells. Red blood cells lysis was performed before labeling with specific antibodies and immune phenotyping with BD FACS Canto II flow cytometry (BD Biosciences, Erembodegem, Belgium). Mice were sacrificed by cervical dislocation at specific time points to evaluate engraftment of human cells in different lymphoid organs of the CD34^+^ transplantedanimals. Lymph nodes, spleen, bone marrow and thymus were dissociated with syringes to obtain single-cell suspensions and passed through a nylon cell strainer, washed three times with RPMI-1640, labeled with antibodies and analyzed for the presence of different human cell populations.

Antibodies used were allophycocyanin conjugated anti–human CD45, fluoresceinisothiocyanate (FITC) conjugated anti-mouse CD45, anti–human CD4, anti-human granzymeB, anti-human CD11c, phycoerythrin (PE) conjugated anti–human CD3, anti-human CD69, anti-human HLA-DR, anti-human PD-1, anti-human CD27, anti-human perforin, peridinin chlorophyll protein conjugated (PerCP) anti–human CD8 and anti-human CD14. All antibodies were purchased from BD Biosciences (Erembodegem-Aalst, Belgium). We also used anti-human CXCR4-PE and anti-human CCR5-PE from RD Systems (Oxon, United Kingdom).

In some experiments, white blood cells were numerated using a Cell-Dyn 3700 analyzer (Abbott, Wiesbaden, Germany) with human settings.

### Immunohistochemistry

Immunohistochemistry was performed on tissue sections from thymus, spleen, and lymph nodes of humanized NSG mice. Sections were fixed with 4% paraformaldehyde and dehydrated with graded alcohol. After treatment with heated citrate buffer for antigen retrieval, sections were blocked for endogenous peroxidase activity. Following this, sections were incubated in 10% goat serum and then with primary antibodies at 4°C overnight. Fixed samples were stained with the following antibodies, anti–human CD45 (MS355P, Neomarkers), CD3 (ab828, abcam), CD20 (L26; M0755, DAKO), CD138 (MCA681H, Serotec), Ki67 (MIB1; M7240, DAKO), and HIV-1 Gag p24 (DAKO) for detection of infected cells. After incubation for 30 minutes with the secondary antibody, the specimens were visualized by DAB treatment. Sections were lightly counterstained with hematoxylin to enable visualization of nuclei. Stained specimens were observed by using transmitted light microscopy (Olympus BX 40, Aartselaar, Belgium).

### Proliferative Responses of Lymph Node and Spleen T-Cells Ex Vivo

Single-cell suspensions of mononucleated cells were prepared from the spleens and lymph nodes of mice after 22 weeks of CD34^+^ cell transplantation. PBMCs from healthy human donors were also isolated by centrifugation over Ficoll-Hypaque (Lymphoprep; Nycomed, Birmingham, United Kingdom). Cells were washed in RPMI-1640 after treatment with erythrocyte lysis buffer and then suspended in RPMI-1640 complete medium supplemented with 10% (v/v) heat-inactivated FBS. These cells were cultured at 1×10^6^/ml (100 µl/well), at 37°C in a 5% CO_2_ and humid atmosphere in round-bottom 96-well microtiter plates (Costar, Kruibeke, Belgium), with medium alone or with the combination of immobilized CD3 (10 µg/ml) (145-2C11, BD Biosciences) and anti-CD28 (1 µg/ml) (CD28.2, BD Biosciences) or anti-CD3 (10 µg/ml) and recombinant IL2 (50 U/ml) (Roche, Vilvoorde, Belgium). After culture cells for 68 h, cells were pulsed with 0.4 µCi/well ^3^H thymidine and incubated for an additional 4 h before harvesting. Cells were harvested onto glass fiber filters (Filter Mate, PerkinElmer, Zaventem, Belgium), and radioactivity was quantified using TopCount® NXT™ Microplate Scintillation and Luminescence Counter (PerkinElmer, Zaventem, Belgium). Samples were assayed in triplicate. The data are presented as mean cpm±standard deviation (SD).

### Virus Culture

The HIV-1JRCSF and HIV-1Bal were obtained from the NIH-AIDS Research and Reference Reagent Program. PBMCs were isolated from HIV-1 seronegative individuals and cultured in RPMI-1640 supplemented with 10% FBS and antibiotics with 5 µg/ml of phytohemagglutinin for 3–4 days (PHA-PBMCs). Cells were then mixed with the virus and cultured for seven days in RPMI-1640, supplemented with 20% FBS, IL2, polybrene and antibiotics. The endpoints were determined by screening for the p24 antigen using ELISA (Innogenetics NV, Ghent, Belgium).

### HIV-1 Infection

Infection and maintenance of humanized NSG mice were performed in GIGA-R-Biosafety Level 3 facilities under standard caging conditions. 130 to 155 days post CB-CD34^+^ cell transplantation, two groups of seven mice each were inoculated i.v. with HIV-1JRCSF (10,000 TCID50) and HIV-1Bal (10,000 TCID50). Plasma from infected mice were collected six times at 20-day intervals to determine HIV-RNA copy number. Blood cells were prepared after RBC lysis to measure CD4+T cell counts using flow cytometry. We also inoculated some mice (n = 3) through rectal route. These mice were allowed to defecate before rectal infection to prevent the immediate elimination of the virus. Infections were performed in a volume of 75 µl (2500 TCID50) of HIV-1Bal. Sterile 200 µl tips were preheated over a flame to smoothen abrasive and sharp surfaces and were then used to deliver the virus. Following delivery of the virus mice were kept in an inverted position for 5 minutes to help virus adsorption in the rectum.

### Viral load Determination

Plasma viral RNA copy numbers were measured using cobas ampliPrep/cobas TaqMan HIV-1 Test, version 2.0 (v2.0) (Roche, Vilvoorde, Belgium). It is an *in vitro* nucleic acid amplification test for the quantitation of HIV-1 RNA in plasma using the cobas ampliPrep instrument for automated specimen processing and the cobas TaqMan analyzer for automated amplification and detection. We processed five to seven samples in each group per time point. Fifty microliters of murine plasma samples were diluted with 950 µl of assay diluent and processed with control seropositive and seronegative human plasma samples. To determine proviral loads, DNA was isolated using an extraction buffer (50 mM KCl, 10 mM Tris-HCl pH 8.3, 2.5 mM MgCl_2_, 0.5% Tween 20, NP40 and Proteinase K). Earlier published HIV *pol* and *gag* gene primers (Table 1) [16], [17] were used to detect provirus in peripheral blood of infected humanized mice by PCR. PCR was carried out in a 100 µl reaction mixture containing 50 mM KCl, 10 mM Tris-HCL pH 8.3, 2.5 mM MgCl_2,_ 0.2 mM dNTP, 2.5 units of Taq DNA polymerase (Roche, Vilvoorde, Belgium), 0.4 µM of each primer and 10 ng of DNA. First round of amplification was performed with outer primers for 35 cycles, then 2 µl of the amplified DNA were amplified for 25 cycles with inner primers. DNA from 8E5/LAV cells (NIH-AIDS Research and Reference Reagent Program) was used as positive control.

**Table 1:** Primers used for *gag* and *pol* gene DNA identification in the peripheral blood of HIV-1 JRCSF infected humanized NSG mice.

Region	Primer code	Sequences(5′-3′)
*pol*	HPOL 4235*	outer 5′-CCCTACAATCCCCAAAGTCAAGG-3′
	HPOL 4538*	outer 5′-TACTGCCCCTTCACCTTTCCA-3′
	HPOL 4327*	inner 5′-TAAGACAGCAGTACAAATGGCAC-3′
	HPOL 4481*	inner 5′-GCTGTCCCTGTAATAACCG-3′
*gag*	HIV-gag1*	outer5′-GCATTATCAGAAGGAGACCACCCCACAG-3′
	HIV-gag2*	outer5′- TCCTGAACGGTACTAGTAGTTCCTGCTA-3′
	HIV-gag SK145*	inner 5′-GGTACATCAGGCCATATCACC-3′
	HIV-gag SK150*	inner5′-ACCGGTCTACATAGTCTC-3′

### Evaluation of Humoral Immune Responses

Concentrations of total human IgM and IgG in plasma of humanized NSG mice were determined before and after HIV-1 infection by conventional human immunoglobulin quantification, ELISA assay (Zeptometrix Cor., Buffalo, USA). HIV specific IgG and IgM human antibodies were detected in the plasma sample from HIV infected humanized mice by line immunoassay using INNO-LIA™ HIV I/II Score (Innogenetics NV, Ghent, Belgium) according to the manufacturer’s instructions. Human positive and negative controls were included with mice samples. 10 µl of sample was added to 1 ml sample diluent, test strips were completely submerged in it and incubated overnight at room temperature on a shaker. After washing three times in wash solution, strips were incubated with the substrate solution, and the reaction was stopped by stop solution. Results were interpreted after the strips were dry.

### Evaluation of Soluble Markers of Non-Specific Immune Activation

β_2_-microglobulin levels were measured in mice plasma samples before and after HIV-1 infection using an ELISA kit (Beckman Coulter, Woerden, Netherlands). Lipopolysaccharide (LPS) levels were also measured in mice plasma samples with standard ELISA kits (Hyglos GmbH, Bernried, Germany and eBioscience, Frankfurt, Germany).

### Enzyme-Linked Immunosorbent Spot Assays

HIV-specific human T cells were identified in mononuclear cells pooled from the lymph nodes and spleen of individual mice. Enzyme-linked immunospot (ELISPOT) assayswere performed using 2×10^5^ cells/well with peptide pool in 96-well polyvinylidene plates (Millipore, Molsheim, France) pre-coated with capture anti–human IFN-γmAb (Diaclone Besancon, France) and incubated at 4°C overnight. Next day cells were seeded in the presence of proper stimulant (SEB or peptide pools). Plates were incubated overnight at 37°C in 5% CO_2_ and developed according to the manufacturer’s recommendations. Spots were counted using an automated ELISPOT reader (Zeiss, Le Pecq, France). Peptide pools consisting of 20 mers, with 10 amino acid overlaps and spanning all expressed HIV proteins in the consensus clade B sequences ofenvgp120s andgag p24, were obtained from the National Institute for Biological Standards and Controls, United Kingdom.

### Statistical Analysis

Statistical analysis was performed using Graphpad Prism software. Data were expressed as the mean value ± SD. Significant differences between data groups were determined by student t-test analysis. *P* value less than 0.05 was considered significant.

## Results

### Human Immune Cell Reconstitution in NSG Mice

**Figure 1:**
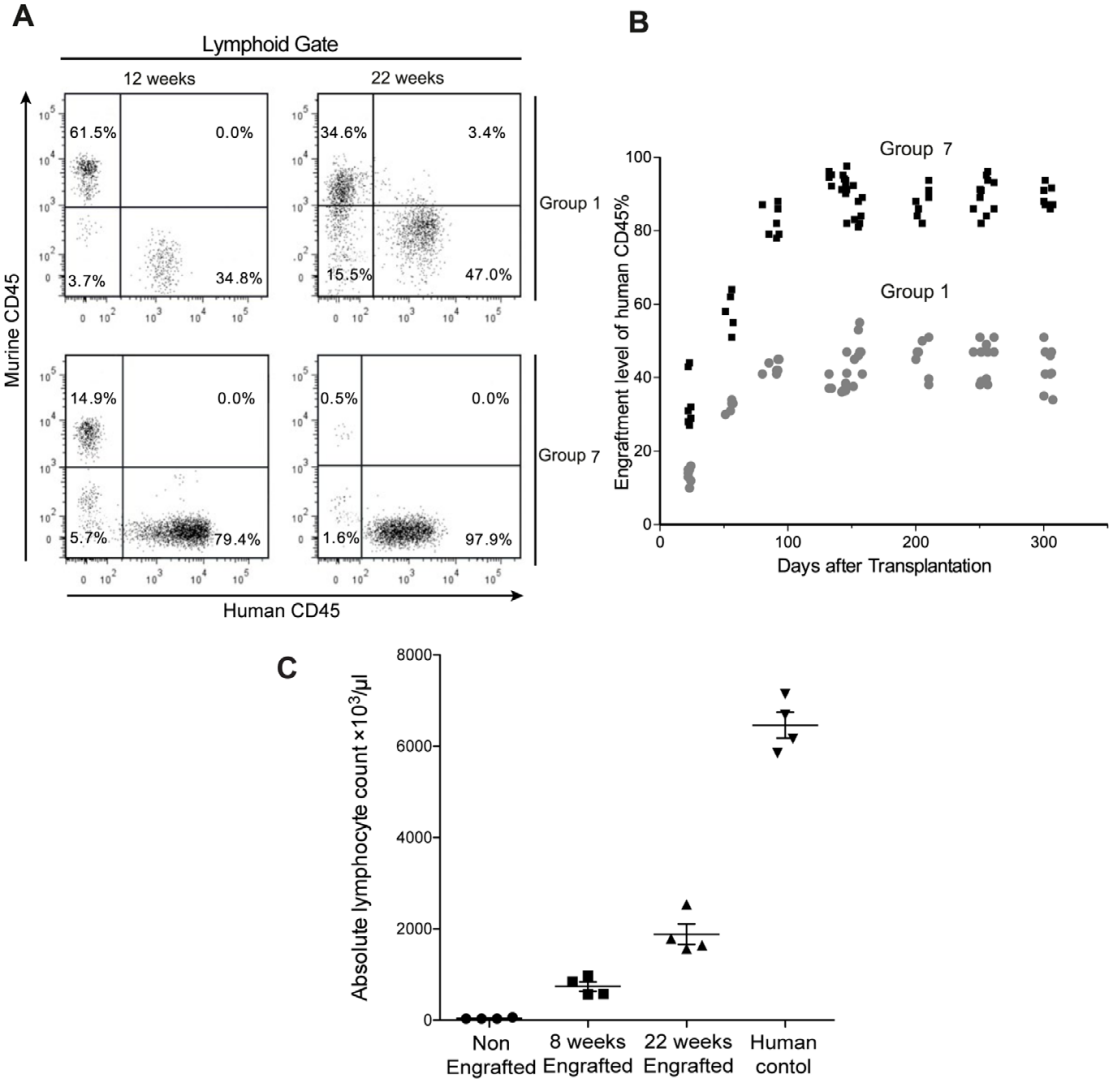
Engraftment potential of CD34^+^ hematopoietic cells. (A) Flow cytometric analysis of murine and human CD45^+^ cells in the peripheral blood of NSG mice of two different groups, representative profiles of the mice engraftment levels after 12 and 22 weeks after CD34^+^ cells transplantation. (B) Engraftment levels of human CD45^+^ cells in peripheral blood up to 306 days after transplantation in group 1 and group 7. (C) White blood cell counts at 12 and 22 weeks post-engraftment in group 7 mice along with human control.

Previous protocols for humanization of NSG or NOG mice required TBI before transplantation of CB-HPCs in order to obtain sufficient CD45^+^ human cell engraftment [5], [6], [7]. Alternative protocols using busulfan as a myelosuppressive agent in neonate NSG mice have also been described [12], [13], [14], [15]. Although busulfan indeed improves human CD45^+^ cell engraftment levels in humanized NSG mice, some limitation persists in the published protocols (i.e. need to transfer large numbers of CB–CD34^+^ cells, cost of cytokine treatment, difficulty of i.v. injections in mice neonates and limited availability of human fetal tissue) [12], [14], [15]. With the goal to improve busulfan-based humanization protocols, we first treated a group of twenty 8–9 week old mice (group 1) with i.p. injection of busulfan 20 mg/kg, 24 hours prior to i.v.transplantation with 1×10^5^ frozen CB-CD34^+^ cells. We then evaluated engraftment levels by iterative analysis of the blood of engrafted mice. We did not observe any mortality with this dose of busulfan. Engraftment levels of human CD45^+^ cell at week 22 were 41.26±5.70% (n = 20) and stable engraftment persisted up to 300 days (Fig. 1A and 1B).

We then sought to determine if changing transplantation parameters could further improve engraftment of human CD45^+^ cells. In a second group of twelve 8–9 week old mice (group 2), busulfan was used at 30 mg/kg in combination with 1×10^5^ frozen human CB-CD34^+^ cells. This regimen allowed to reach engraftment levels of 47.61±4.10% (n = 12), significantly better than with busulfan 20 mg/kg (group 1) (*P* = 0.0021). Decreasing the age of mice to 5–7 weeks and using the same dosage of busulfan (30 mg/kg) further improved the engraftment to 51.13±6.13% (n = 6) (group 3), but this gain was not statistically significant in comparison with group 2. Next, we increased the number of transplanted CB-CD34^+^ cells from 1×10^5^ to 2×10^5^. At the dosage of 30 mg/kg busulfan (group 5), the engraftment was 64.45±3.10 (n = 7) and was very significantly better than with 1×10^5^ cells (group 2) (*P*<0.0001) (Table 2). We also evaluated 100-cGy irradiation and intrahepatic injection of 1×10^5 ^CB-CD34^+^ cells in neonatal mice (n = 10) (group 6), but observed 100% mortality within seven days of transplantation.

**Table 2:** Transplantation conditions and engraftment levels of human CD45^+^ cells after 22 weeks in various groups of NSG mice.

Group Number	Age of mice	Busulfan dose	CB-HSCs	CD45 Engraftment %
Group 1, n = 20Group 2, n = 12Group 3, n = 6Group 4, n = 6Group 5, n = 7Group 6, n = 10Group 7, n = 20Group 8, n = 18	8–9 weeks8–9 weeks5–7 weeks8–9 weeks8–9 weeks5–7 days3–4 weeks3–4 weeks	20 mg/kg30 mg/kg30 mg/kg20 mg/kg30 mg/kgTBI-100cGy25–25 mg/kg(split dose)30–30 mg/kg(split dose)	Frozen−1×10^5^Frozen−1×10^5^Frozen−1×10^5^Frozen−2×10^5^Fresh−2×10^5^Frozen−1×10^5^Fresh−2×10^5^Fresh−2×10^5^	41.26±5.70%47.61±4.10%51.13±6.13%,48.33±5.13%,64.45±3.10%−91.97±4.10%−

Careful observation of the various protocols (Table 2) reveals significant improvements of human CD45^+^ cell engraftment by increasing the dose of busulfan to 30 mg/kg (group 5) and using 2×10^5^ fresh CD34^+^ cells. Therefore, in order to further optimize the transplantation protocol, we concentrated on these parameters.

Neonatal mice classically show better engraftment compared to adults but injecting them with HPCs is a delicate work. In contrast, weaning and i.v. tail injection of 3–4 week old mice is an easier procedure. Therefore, we chose this age group and used fresh rather than frozen CB-CD34^+^ cells. Hayakawa *et al*. reported good tolerability of 50 mg/kg busulfan conditioning in NSG mice. Hence we decided to administer two doses of busulfan 25 mg/kg with a 12-hour interval. Under these conditions, the percentage of human CD45^+^ cells at week 22 was 91.97±4.10% (n = 20) and mice survived up to 300 days (group 7) (Fig. 1A and 1B). This level of engraftment was very significantly better than in group 5 (*P*<0.0001). We also measured white blood cell counts in the blood of group 7 mice at 8 and 22 weeks after CB-CD34+ cell transplantation. At 22 weeks, white blood cell count was 1885±44 cells/µl for humanized mice and 6462±285 cells/µl (n = 4) in human controls analyzed with the same settings (Fig. 1C). A further increase in the busulfan dosage to 60 mg/kg caused complete lethality (n = 18) (group 8).

### Transplantation of Fresh CB CD34^+^ Cells Eesults in the Differentiation of Major Human Leukocyte Lineages in Blood and Lymphoid Organs

**Figure 2:**
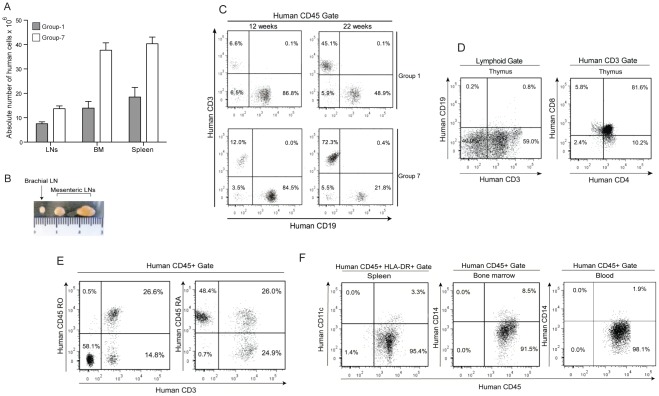
Human cell differentiation in humanized NSG mice. (A) Absolute number of human cells in lymph nodes, bone marrow and spleens of group 1 and group 7 mice. (B) Mesenteric and brachial lymph nodes from male mice obtained after 24 weeks of CD34^+^ cell transplantation in group 7 mice. (C) Flow cytometric analysis of engraftment percentage of CD3^+^ T cells and CD19^+^ B cells at 12 and 22 weeks after transplantation of CD34^+^ cells in representative mice from group 1 and group 7. (D) Flow cytometric analysis of CD4^+^CD8^+^, CD4^+^ and CD8^+^ population in thymus of 22 weeks engrafted mice. (E) Flow cytometric analysis of CD45RA^+^ and CD45RO^+^ positive CD3 cells 22 weeks after engraftment in mice from group 7. (F) Representative profile CD11c^+^ cells in spleen and CD14^+^ population in bone marrow and blood of group 7 mice 22 weeks after CD34^+^ cell transplantation.

We next evaluated if the overall improvement of human cell engraftment associated with group 7 protocol was observed in all lymphoid sites and specifically correlated with a better or faster differentiation of a given cell subset (*i.e.* CD19, CD3, CD4, CD8). We compared engraftment achieved in groups 1 and 7, in blood, spleen, lymph nodes and bone marrow at 12 and 22 weeks after transplantation of CB-CD34^+^ cells. At 22 weeks, high percentages of human CD45^+^ cells were observed in the lymph nodes (95.51±3.23%), bone marrow (83.41±2.65%) and spleen (74.31±2.92%) (n = 20) (Table 3). Humanized mice of group 7 also had higher absolute numbers of human cells in lymph nodes, bone marrow and spleen compared with group 1 (Fig. 2A). Twenty-four weeks after transplantation of fresh CB-CD34^+^ cells, lymph nodes of sizes from 3 to 7 mm could be observed in NSG mice from group 7 (Fig. 2B).

**Table 3:** Engraftment levels of human cells in peripheral blood (PB), spleen, lymph nodes (LNs) and bone marrow (BM) of humanized NSG mice.

	Weeks afterCD34+ cellstransplantation	12-Weeks						22-Weeks					
	Cell Percentage	% Murine CD45+	% Human CD45+	% CD19^+^ofhumanCD45	% CD3^+^ofhumanCD45	% CD4^+^ofhumanCD45	% CD8^+^ofhumanCD45	% Murine CD45	% Human CD45	% CD19^+^ofhumanCD45	% CD3^+^ofhumanCD45	% CD4^+^ofhumanCD45	% CD8^+^ofhumanCD45
PB	Group 1Group 7	71.40±6.70%	28.26±8.70%	87.60±3.47%	4.52±2.08%	1.43±0.98%	1.69±0.68%	58.74±7.70%	41.26±5.70%	51.60±4.21%	35.60±5.87%	23.43±0.98%	12.69±2.78%
		21.74±3.79%	78.26±6.89%	79.32±3.18%	13.45±1.90%	4.13±1.78%	6.91±2.18%	8.03±2.70%	91.97±4.10%	22.52±3.08%	67.29±4.88	48.81±6.78%	19.39±4.13%
Spleen	Group 1Group 7	78.34±4.07%	21.66±3.70%	91.90±4.51%	3.52±0.88%	1.11±0.98%	1.89±0.68%	68.34±8.10%	31.66±4.30%	68.60±5.21%	20.91±6.17%	8.43±2.08%	12.69±1.18%
		41.74±3.07%	58.26±3.99%	83.32±4.18%	10.15±1.91%	3.03±0.13%	7.12±1.17%	25.69±2.17%	74.31±5.92%	52.52±6.88%	42.29±4.88%	16.81±6.85%	12.39±4.43%
LNs	Group 1Group 7	−	−	−	−	−	−	24.84±3.30%	75.16±13.30%	38.60±6.51%	50.66±3.87%	24.03±4.98%	14.69±2.78%
		11.84±2.31%	88.16±7.09%	73.11±4.18%	21.15±3.90%	7.53±1.78%	12.99±3.18%	4.49±0.70%	95.51±3.23%	21.52±2.98%	77.29±11.88	48.81±6.18%	21.09±4.43%
BM	Group 1Group 7	74.67±6.07%	25.33±7.70%	85.11±6.47%	3.33±1.18%	0.83±0.99%	1.89±0.78%	61.14±11.0%	38.86±4.45%	54.10±3.23%	40.60±4.87%	22.43±3.98%	16.69±2.78%
		32.84±4.10%	67.16±8.99%	74.13±4.41%	11.23±2.90%	4.13±1.33%	5.91±11.88%	16.59±3.10%	83.41±2.65%	40.32±10.08%	55.29±5.18	38.81±6.78%	14.39±4.13%

Peripheral blood and organs were collected at 12 and 22 weeks after CD34^+^ cell transplantation in group 1 and group 7 mice. Percentage of human and murine CD45 cells was calculated on lymphoid gate. Percentage of human CD19^+^, CD3^+^, CD4^+^ and CD8^+^ cells were calculated out of human CD45^+^ cells.

In both groups, CD19^+^ B cells were the major subset present at week 12 (representing more than 80% of human CD45^+^ cells) and gradually declined afterwards. The decline of B cells was more pronounced in group 7 in consistence with the T cell enrichment associated with this protocol (Table 3 and Fig. 2C).

In accordance with these results, we found good proportions of CD3^+^ T cells in mice from group 7. At week 12, the percentage of CD3^+^ T cells was roughly three times higher in group 7 than in group 1, all sites considered (Table 3 and Fig. 2C). Mesenteric lymph nodes that were virtually absent in mice from group 1, were well characterized in mice from group 7 and yielded a high proportion of T cells (21.15±3.90%, n = 5). At week 22, the percentage of CD3^+^ T cells in the blood of mice from group 7 was 67.29±4.88% (n = 20) and remained significantly higher compared to group 1 (35.60±5.87%, n = 20) (Table 3 and Fig. 2C). Similar results were obtained for spleen and bone marrow. Interestingly, engraftment was very good in thymus with 98.63±1.23% human CD45^+^ cells and 61.10±4.91% (n = 3) human CD3^+^ thymocytes. Among CD3^+^ cells, 82.13±3.43% were CD4^+^CD8^+^ double positive and smaller proportions were single positive CD4^+^ (8.71±2.13%) and CD8^+^ (5.61±3.21%) cells (Fig. 2D). Among the CD3^+^ T cell population of peripheral blood of humanized mice 52.12±11.11% (n = 4) were CD45RO and 46.43±13.11% were CD45RA (n = 4) (Fig. 2E).

Human monocytes, identified by the expression of human CD14 were observed in the bone marrow (8.22±3.16%) and blood (2.31±0.83%), 22 weeks after engraftment of mice from group 7 (Fig. 2F). With the same protocol, human myeloid DCs identified by the expression of CD11c were also present in the spleen (3.12±1.21%, n = 6) (Fig. 2F).

### Expression of HIV-1 Coreceptors

**Figure 3:**
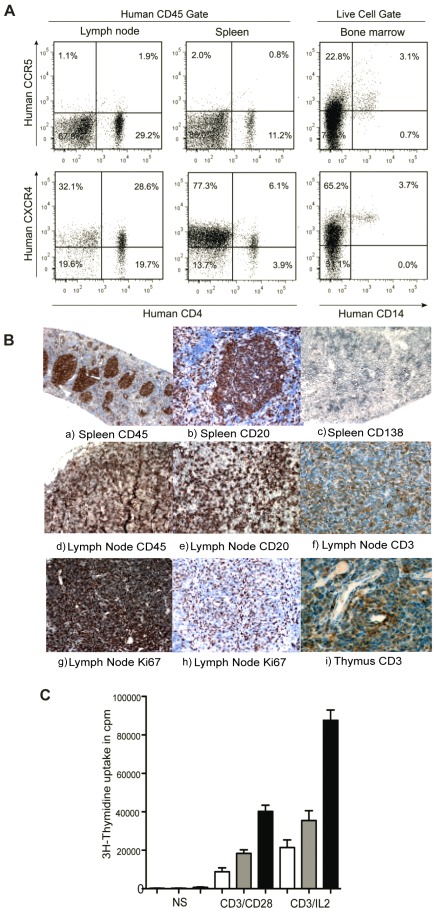
Coreceptor expression and lymphoid organ formation in humanized mice. (A) Representative FACS profile of human CCR5^+^CD4^+^ and CXCR4^+^CD4^+^ cells in spleen and lymph nodes, gate was set of human CD45 cells. Expression of CCR5 and CXCR4 were checked on CD14^+^ cells, gate was set on live human cell population. (B) Histology of lymphoid organs in CD34^+^ engrafted NSG mice. The lymphoid follicles mainly contained hCD45 cells. Spleen sections were stained with (a) anti-hCD45, (b) anti-hCD20 and (c) anti-hCD138. Lymph node sections were also stained with (d) anti-hCD45, (e) anti-hCD20 (f) anti-hCD3 (g,h) anti-hKi67 antibodies. Thymus section was stained with anti-CD3 antibody. (C) Proliferative responses of T cells were measured in nonstimulated cells, after stimulation with immobilized anti-CD3/anti-CD28 and anti-CD3+IL2 in spleen (white bars), lymph nodes (gray bars) and human PBMCs (black bars).

Next we studied the expression of HIV-1 co-receptors CXCR4 and CCR5 on human CD4^+^ T cells in the lymphoid organs of the humanized mice. CXCR4 was expressed on 78.11±17.13% (n = 4) CD4^+^ T cells in spleen and on 42.1±14.1% (n = 4) of CD4^+^ T cells in the lymph nodes (Fig. 3A). The proportion of CD4^+^ T cells positive for CCR5 was lower in both lymph nodes 6.51±2.13% and spleen 7.17±3.133%, indicative of a naïve state of T cells (Fig. 3A). Large proportions of CD14^+^ cells in the bone marrow were also positive for CCR5 and CXCR4 (Fig. 3A).

### Formation of Lymphoid Structures

After 22 weeks of CD34^+^ cell transplantation in NSG mice, we investigated the lymphoid structure formation and development of human leukocytes, which is essential for elicitation of immune responses against foreign antigens and the spread of HIV-1 infection. Immunohistochemical analysis of human CD45 leukocytes showed that they gathered in a form of follicle-like structures (FLSs) at the end of the central artery in the spleen. In serial sections of the same region, these structures consisted mainly of human CD20^+^ B cells admixed with a small number of human CD138^+^ plasma cells. High levels of human CD45 cells in lymph nodes were also confirmed by immunohistochemistry. Labeling with CD3 and CD20 markers confirmed the presence of T and B cells in lymph nodes. Lymph nodes have high levels of Ki-67 positive cells compared with the spleen, suggesting the presence of high levels of actively proliferating cells. Also a high percentage of human CD3^+^ cells could be seen in the thymus of engrafted mice (Fig. 3B).

### TCR-Mediated Proliferative Responses of Human T Cells Differentiated in a Murine Environment

Next, in order to prove that human T cells originating from murine thymopoïesis are indeed functional, we measured proliferative responses induced by an antibody directed against human CD3 in mononucleated cell suspensions prepared from murine spleen and lymph nodes 22 weeks after transplantation of CB-CD34^+^ cells and compared them with those observed with PBMCs from healthy donors. Although lower than in PBMCs, significant proliferative responses were observed in cells isolated from both murine lymphoid organs (Fig. 3C). The responses were higher in lymph nodes than in spleen (Fig. 3C) especially when IL-2 rather than anti-CD28 was used as a cosignal (Fig. 3C). Since the proportion of human T cells differs between the different cell preparations, this non-standardized assay does not allow to interpret the differences observed but clearly rules out a global anergy of T cells recovered from humanized mice.

### Sustained, High-Level of HIV-1 Replication in Humanized NSG Mice

**Figure 4:**
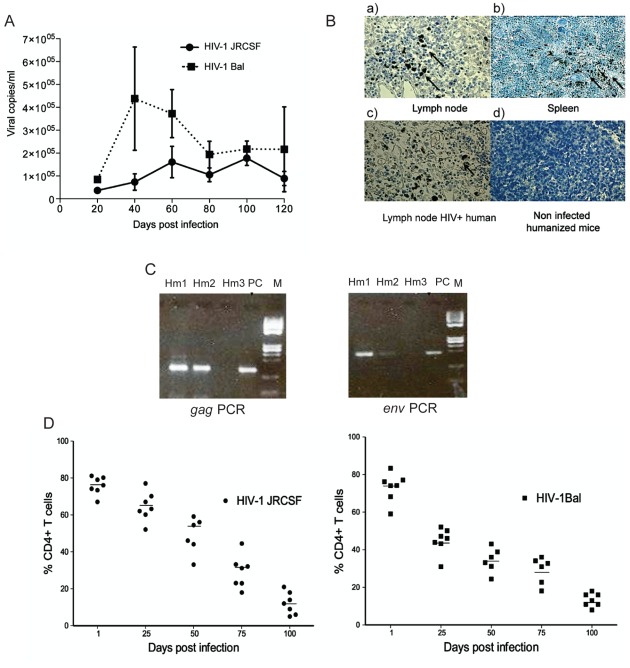
Sustained high-level of HIV dissemination and infection progression. (A) Humanized mice were i.v. injected with HIV-1 JRCSF and HIV-1 Bal at 10,000 TCID. Mice were bled at 20, 40, 60, 80, 100 and 120 days to detect plasma viral load, curve represent the viral load at different time point. Error bars (B) Sections of lymph node (a) and spleen (b) of humanized HIV infected mice were stained using anti-p24 antibody. Brown color show p24 specific staining. Lymph node of HIV-1 infected human (c) is shown as positive control and engrafted noninfected mouse spleen (d) is shown as negative control. (C) DNA was extracted from peripheral blood of HIV-1JRCSF-infected mice 9 week after infection. Determination of HIV-1 DNA copy was performed by PCR assay for *gag* and *pol* gene. 8E5 cell DNA was amplified as positive control (PC) along with humanized mice DNA (hm1, hm2, and hm3) (D, E) Percentage of CD4^+^ cells of T-cells following HIV-1 infection, each circle represent one mouse infected with HIV-1 JRCSF and each square represent one mouse infected with HIV-1 Bal means of CD4% of CD3^+^ cells at time point of infection are shown in solid line.

Humanized mice were inoculated i.v. with two different HIV-1 isolates. We usedCCR5-tropic (R5-tropic) JRCSF and HIV-1Bal isolates inoculated at 10,000 TCID50 doses for infection. Mice were bled at 20, 40, 60, 80, 100 and 120 days postinfection and plasma viral loads were determined by cobas AmpliPrep/cobas Taq Man Version 2.0 HIV-1 assay. Productive infection was observed after inoculation with both isolates. For HIV-1Bal, viral load reached 4.2×10^5^±2.2×10^5^ copies/ml on day 40 and remained over 1.9×10^5^±5.8×10^4^ copies/ml at the three following time points. Viral loads were lower for HIV-1JRCSF reaching 1.6×10^5^±3.5×10^4^ copies/ml at day 60 and 1.85×10^5^±3.2×10^4^ copies/ml 100 days after infection. Viral loads were still detected at day 120 for both isolates (Fig. 4A). In a preliminary experiment, mice infected by the rectal route (BAL isolate at 2500 TCID50) and showed high levels of plasma viral load 0.93×10^5^±2.2×10^4^ copies/ml (n = 3) 35 days after inoculation.

We confirmed the presence of HIV in the peripheral lymphoid tissues of humanized infected mice by immunohistochemical staining of HIV gag-p24 antigen. Infection was distributed primarily in the T cell rich periarteriolar lymphoid sheaths of the spleen and was more diffuse in the lymph nodes of humanized mice (Fig. 4B). A necropsic section of the lymph node of a 39 year old patient deceased of AIDS in 1990 was provided by the Liège Biobank (Ethical agreement 2010/29) and stained for gag p24 as a positive control. Detection of proviral DNA was also performed to in these humanized NSG mice. We isolated DNA from circulating peripheral blood of mice nine weeks postinfection and performed *gag* and *pol* gene PCR. We could detect provirus in five mice out of nine. Three mice were positive for *gag* and two were positive for *pol* (Fig. 4B). We could not detect proviral load in all the samples either because of PCR failure or peripheral blood cells might have lower levels of proviral DNA.

### HIV-1 Infection Progression in Infected Humanized NSG Mice

Infections with both HIV-1JRCSF and HIV-1Bal isolates were associated with depletion of CD4^+^ T cells in the blood of humanized NSG mice. Decline in CD4^+^ T cell percentage could already be seen at 25 days after infection and continued until 120 days postinfection (4 to 6 mice each timepoint) (Fig. 4D and 4E). We have measured total numbers of circulating lymphocytes at nine time points in three HIV-Bal infected humanized mice to determine the absolute numbers of CD4^+^ T cells. The absolute CD4^+^ cell count decreased significantly to the same extent as the percentage CD4^+^ T cells declined. Before infection three infected mice had average CD4 count of 672±96.08 cells/µl, but it decreased significantly (493.33±72.34 and 358.66 cells/µl) 25 and 50 days after infection. However, because of restriction of sample volume to perform dual platform method analysis (hematological analyzer and flow cytometry), determination of absolute CD4^+^ cell count could not be performed at all-time points.

### Immune Activation, Exhaustion and Senescence after Infection of Humanized NSG Mice with HIV-1JRCSF

**Figure 5:**
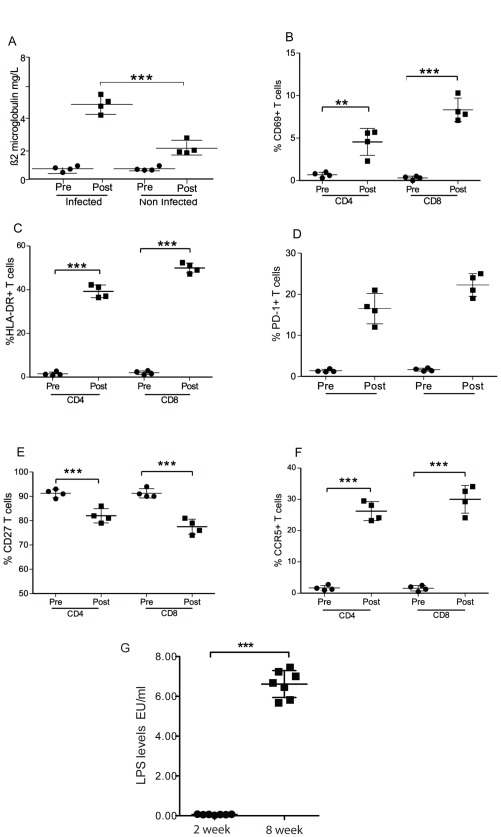
HIV induced immune activation and expression levels of multiple activation markers on CD4^+^ and CD8^+^. Blood samples were obtained from mice before and 6 week after HIV-1 JRCSF infection. (A) Beta-2-microglobline levels were measured using ELISA before (Pre) and after HIV-infection (Post) (n = 4) and at same time intervals in engrafted but noninfected mice. (B) CD69 (C) HLA-DR (D) PD-1 and (E) CD27 (F) CCR5 levels were evaluated on CD4^+^ and CD8^+^ cells. (G) LPS levels in humanized mice 2 and 8 weeks post-HIV infection. Each circle and square represent one mouse at the indicated time point. Means are shown in solid lines. *P* values were determined by unpaired student’s t-tests. ** indicates a *P* value<0.001 and *** indicates a *P* value<0.0001.

Aspecific activation of the innate immune system has been described in HIV-1 infection and possibly drives subsequent activation of the adaptive immune response. Levels of non-specific immune activation marker such as β_2_-microglobulin have been correlated with disease progression [18]. In humanized mice, HIV infection induced a strong increase of β_2_-microglobulin from 0.42±0.09 mg/l at day 0 to 5.41±0.29 mg/l (n = 4). Aging in non-infected mice was associated with a moderate increase of β_2_-microglobulin but this marker remained much lower than in the infected animals (Fig. 5A). Next we studied the expression of early activation markers CD69 and HLA-DR on CD4 and CD8 T cells of humanized mice after HIV infection. The percentage of CD4^+^ T cells expressing CD69 increased from 0.67±0.29% to 4.55±1.58% (n = 4) whereas it went from 0.30±0.21% to 8.33±1.38% (n = 4) for CD8^+^ T cells (Fig. 5B). The increase in HLA-DR expression after HIV infection was even more pronounced. The expression of HLA-DR was observed on 2.50±0.81% of CD4^+^ T cells and 3.00±0.80% of CD8^+^ T cells before infection and jumped to 39.28±2.90% (n = 4) and 49.98±2.24% (n = 4) respectively for CD4^+^ and CD8^+^ T cells after infection (Fig. 5C). Some activation markers are specifically linked to impaired T cells functions. This is the case for the inhibitory receptor PD-1 (Programmed Death 1), which is a marker for T cell exhaustion [19]. After infection with HIV-JRCSF, there was a strong increase of the percentage of T cells positive for PD-1 in comparison with non-infected counterparts. This was true for CD4^+^ (16.50±1.84% (n = 4) *vs.* 1.29±0.89% (n = 4)) as well as for CD8^+^ T cells (22.25±1.37% (n = 4) *vs*. 2.59±2.34% (n = 4)) (Fig. 5D). CD27, a receptor involved in co-stimulation, is down regulated with advancing cell differentiation from early-differentiated CD27^+^ memory T cells to late differentiated CD27^−^ memory T cells. The loss of CD27 on T cells is classically viewed as marker of immune senescence and occurs in HIV infection. In HIV-infected NSG humanized mice, we observed down-regulation of CD27 expression in CD4^+^ (from 91.25±1.70% positive before infection to 82.00±2.94% at week 6) and CD8^+^ T cells (from 91.25±1.89% to 77.50±3.11%) (Fig. 5E).

Activation of CD4^+^ T cells is also associated with upregulation of CCR5 and may therefore render T cells more susceptible to infection. In non-infected humanized mice, only 1.67±0.39% of CD4^+^ T cells and 1.57±0.86% of CD8^+^ T cells were positive for CCR5. Six weeks after infection, 26.23±3.05% of CD4^+^ T cells and 30.03±4.43% of CD8^+^ T cells expressed the HIV coreceptor (Fig. 5F). Microbial translocation has recently been proposed to play a central role in chronic inflammation and subsequent activation of adaptive immunity in HIV-infected individuals [20]. We have measured LPS levels in plasma of HIV-1 infected mice 2 and 8 weeks after infection. At 2 weeks, LPS levels were 0.06±0.02 EU/ml, which increased significantly to 6.62±0.68 EU/ml at 8 weeks after infection (Fig. 5G). LPS was below detection limits in non-infected humanized mice.

### Humoral Immune Responses in HIV Infected Humanized NSG Mice

**Figure 6:**
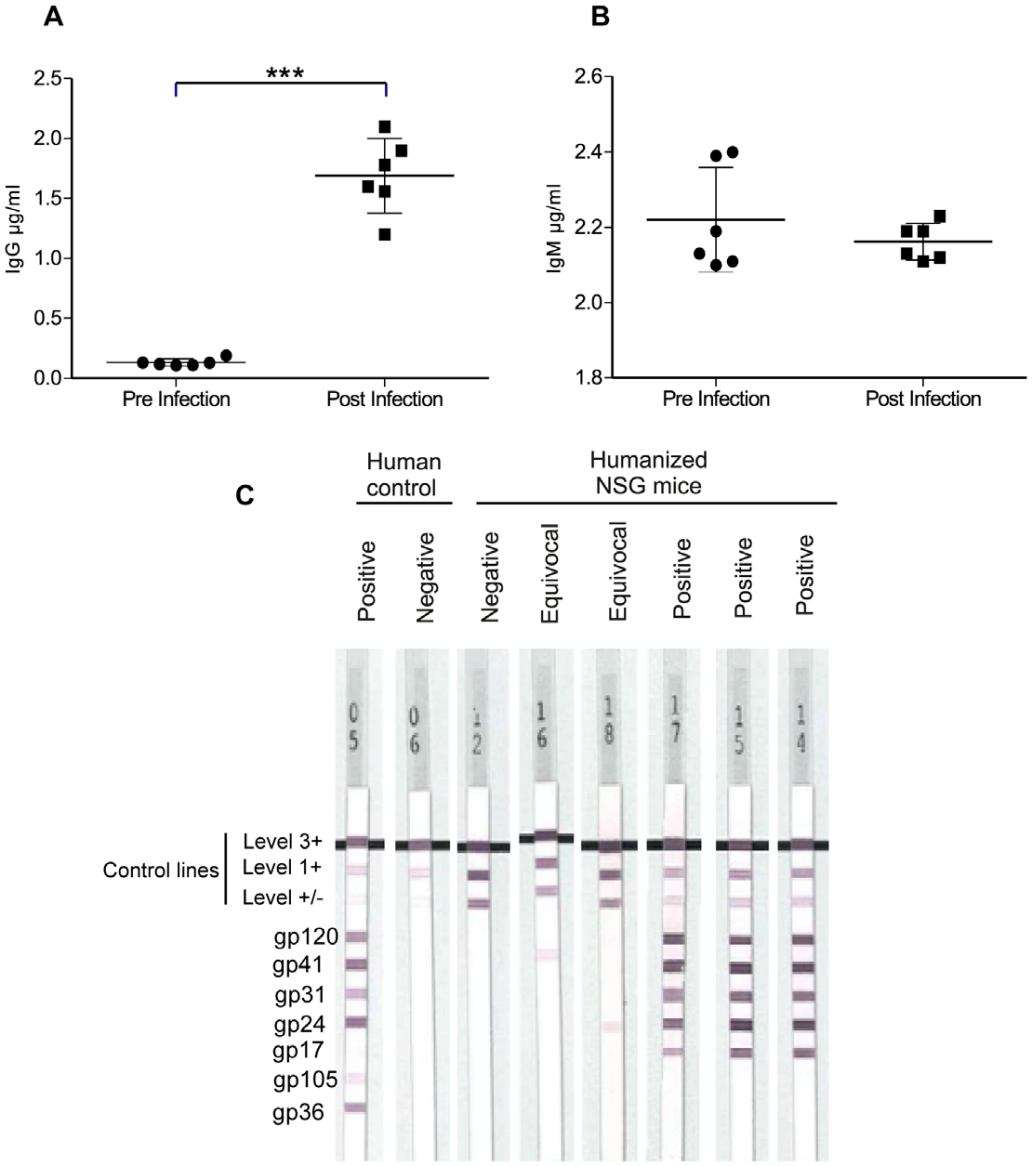
Humoral immune responses in HIV infected humanized NSG mice. (A, B) Detection of IgG and IgM level in the plasma of 6 mice before HIV infection (Pre-closed circle) and 6 weeks after HIV-1 JRCSF infection (Post-closed square) by ELISA assay. Each circle represents one mouse; means are shown in solid lines. *P* values were determined by unpaired student’s t-tests. *** indicates a *P* value<0.0001. (C) Line immunoassay was used to determine HIV-1 specific human antibodies in HIV-1 JRCSF infected mice (n = 12). Nine weeks after infection positive (n = 3) and equivocal (n = 2) anti-HIV-1 specific human antibodies response could be seen. Positive and negative human controls are shown for comparison.

To determine whether B cells are functionally mature and can differentiate into plasmocytes and secrete immunoglobulins after HIV-1 infection, we measured levels of total human IgG and IgM and specific antibodies in humanized mice. Before HIV infection, humanized mice produced low but detectable amounts of IgG 0.13±0.01 µg/ml, n = 6 and IgM 2.22±0.13 µg/ml, n = 6. Interestingly, IgG increased significantly after HIV-1 infection and reached 1.69±0.53 µg/ml, n = 6 (Fig. 6A). Conversely, there was a modest decrease of the IgM levels at 2.16±0.04 µg/ml, n = 6 (Fig. 6B).

We next tested the infected humanized mice for the presence of human antibodies directed against HIV-1, using line immunoassay. Antibody reactions to the HIV antigen bands gp120, gp41, p31, p24, p17 of HIV-1 and gp105 and gp36 of HIV-2 were assessed using INNO-LIA test strips. Six weeks after infection, all mice were negative for anti-HIV-1 specific antibodies. At week 9, 3 mice of out 12 displayed reactivity towards four antigen bands and 2 mice had a weaker response directed against one antigen band only (Fig. 6C).

### Cell-Mediated Immune Responses Directed Against HIV in Infected Humanized NSG Mice

**Figure 7:**
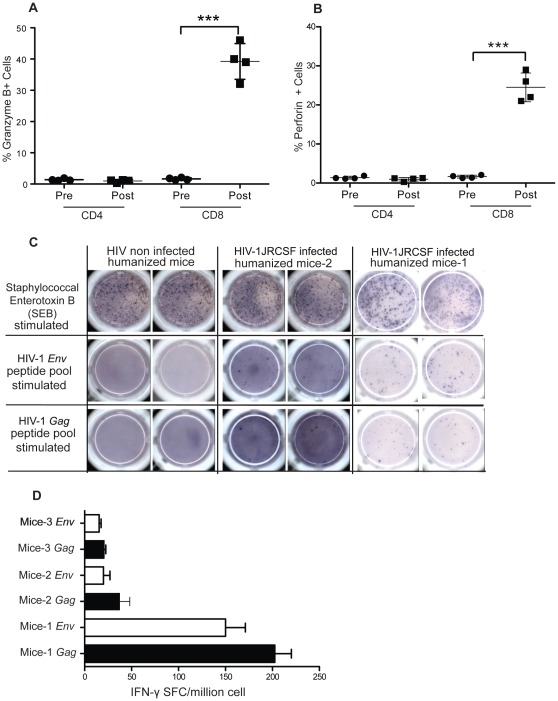
T-cells cellular immune responses to HIV infection. (A, B) Expression of cytotoxic effector molecules perforin and granzyme B are measured by flow cytometry before and 6 weeks after HIV-1 infection in humanized NSG mice (n = 4). Means are shown in solid lines and *P* values were determined by paired student’s t-tests, are shown for significant statistical comparisons. (C) ELISPOT assay measured IFN-γ secretion from mononuclear cells, pooled from lymph nodes and spleen and stimulated with HIV-1 *gag* or *env* peptides. In left panel of engrafted but noninfected mice, non-specific stimulation with SEB lead to generation of response and no IFN- γ secreting cells could be detected after HIV-1 peptide stimulation. In the middle and right panels of HIV infected mice, IFN-γ responses were seen with nonspecific stimulation with SEB but also when stimulated with HIV-1 peptide. HIV-1 Gag peptides stimulation responses were higher than HIV-1 *env* peptides in all infected mice. (D) Quantification of IFN-γ secreting HIV-1-specific T cells in an HIV-1-infected humanized mice detected by an ELISPOT assay. Pools of peptides spanning all of *gag* and *env* were used for overnight stimulation. Each spot represent IFN-γ secreting cell. The frequency of responses is depicted as IFN-γ spot-forming cells per 10^6^ cells.

We also evaluated the generation of specific cellular immune responses directed against HIV antigens. CD8^+^ T cells secreting granzyme B and perforin are critical for efficient cytolytic function. Therefore, we evaluated the presence of these cytotoxic compounds in the CD8^+^ T cells of infected mice using intra-cytoplasmic staining and flow cytometry. We noted a significant increase in the expression levels of both molecules in CD8^+^ T cells six weeks after HIV-1 infection. The percentage of CD8^+^ cells expressing granzyme B increased from 1.65±0.18% (n = 4) to 39.25±2.87% (n = 4) six-week postinfection (Fig. 7A) while the percentage increased from 1.6±0.13% (n = 4) to 24.53±0.81% (n = 4) for perforin (Fig. 7B). Levels of granzyme B and perforin remained unchanged in CD4^+^ T cells (Fig. 7A and 7B).

Next, we performed an IFN-γ-based ELISPOT assay to measure anti-HIV responses in humanized mice nine weeks after infection (n = 5). Mononucleated cells from spleen and lymph nodes of five infected mice were activated with SEB (0.5 µg/ml) as a positive control or with a pool of overlapping peptides for *gag* and *env* (Fig. 7C and 7D). A significant response was observed in 3 out of 5 mice, especially in mouse 1 where 200±13 spot forming cells/10^6^ were counted for *gag* and 150±15 for *env*. Overall responses against *gag* were higher than against *env* in all mice. Two noninfected mice were also tested, and no detectable ELISPOT responses to HIV peptides could be observed (Fig. 7C and 7D).

## Discussion

Development of a long lasting fully functional human immune compartment in immunodeficient mice is a prerequisite for *in vivo* HIV-1 studies [1]. We have developed a practical humanized mouse model that allows, in a reproducible fashion, high levels of human cell engraftment suitable for HIV-1 studies. In our final protocol, we performed conditioning of mice with busulfan at 50 mg/kg administered in two split doses and transplanted fresh CB-CD34^+^ cells in 3–4 week old mice. Our strategy resulted in 92% of human CD45^+^ cell chimerism in the peripheral blood of humanized mice.

We observed significantly higher engraftment levels of human CD45^+^ cells in comparison with previous studies with busulfan-conditioned and CB-CD34^+^ transplanted mice. Hayakawa *et al.* have reported 61% human cell chimerism in humanized mice after 50 mg/kg busulfan preconditioning and transplantation of a high number of CB-CD34^+^ cells (2×10^6^) in 8–12 weeks old mice [12]. Kim *et al.* transplanted CD34^+^ cells along with fetal thymus/liver/bone tissue and observed 31% engraftment of human CD45^+^ cells in peripheral blood of humanized mice. These neonate mice were conditioned with 15 mg/kg busulfan dose. Choi *et al*. have also reported use of 20, 30, and 40 mg/kg busulfan dose for transplantation of CD34^+^ cells in 6-week old NSG mice. However, observed levels of engraftment were lower in these mice compared to our study (Table 4).

**Table 4:** Comparison of published busulfan-preconditioning protocols, kind and number of CD34^+^ cells transplanted and engraftment levels of human CD45^+^ cells in peripheral blood (PB), spleen and bone marrow (BM) of NSG mice.

Busulfan dose	Age of mice	Injection route	Number of cells	Engrafted tissue	CD45 cells percentage			Publication
					PB	Spleen	BM	
15 mg/kg	Newborn	Intrahepatic	CB–CD34^+^, 2×10^5^	−	∼41%	∼61%	∼71%	Choi *et al.* 2011 *Clinical immunology*
15 mg/kg	Newborn	Intrahepatic	FL–CD34^+^, 2×10^5^	Fetal thymus/bone	∼31%	79%	84%	Kim *et al.* 2011 *Journal of clinical immunology*
30 mg/kg	6–8 week	Tail vein	CB–CD34^+^, 1×10^5^	−	62%	80%	45%	Choi *et al.* 2011 *Journal of clinical immunology*
50 mg/kg as (25–25) mg/kg	7–10 week	Tail vein	CB–CD34^+^, 2×10^6^	−	61%	−	−	Hayakawa *et al.* 2009 *Stem cells*

We believe that a unique combination of several factors is likely to explain our good engraftment levels. We were able to use a relatively high dose of busulfan (50 mg/kg) without inducing mortality thanks to the split dose regimen advocated by previous authors [12]. We also used HPCs immediately after their processing by the cord blood bank and without cryopreservation. Unfortunately, our experiments were not set up to discriminate between the respective effect of busulfan dosage on the one hand and using fresh HPCs on the other. Nevertheless, we believe that using HPCs without cryopreservation might be an important factor since previous reports have suggested that cryopreserved-thawed HPCs contain significant proportions of apoptotic cells that do not contribute in engraftment and cannot be detected by regular viability dyes [21]. It is also known that cryopreservation of CD34^+^ cells reduces expression of cell adhesion/homing molecules such as L-selectin (CD62L) which play a major role in the migration of transplanted cells [22]. Further experiments are required to evaluate with more accuracy the importance of this factor in our model.

It is also known that the age of the mice has a significant influence on the engraftment levels of human CD45^+^ cells [8]. We therefore chose to use 3–4 week-old mice, which are furthermore easier to manipulate, compared to neonates.

Another practical advantage of this protocol is the relatively low number of fresh CD34^+^ cells needed to get optimal reconstitution. In comparison with Hayakawa *et al.* we used ten times less CD34^+^ cells to achieve high levels of human CD45^+^ cell engraftment. This allows to engraft more mice with a single cord blood and therefore to limit interdonor variability in functional studies.

The good results obtained at 12 weeks were confirmed at further time points and at different sites including bone marrow, spleen and lymph nodes. Few published reports have described the development of visible lymph nodes in humanized immunodeficient mice [23], [24]. Interestingly, we observe several mesenteric lymph nodes in all our reconstituted mice with sizes ranging from 3 to 7 mm size. We were even able to detect axillary lymph nodes in some engrafted animals.

Importantly, the overall improvement of engraftment levels was associated with the preferential expansion of T cells in these mice (Table 3). Once again, this might be related to the age of the mice used in our experiments. Previous reports have shown differences in thymus architecture between newborn and 8–10 weeks immunodeficient mice [8], [23]. The thymus hypoplastic cysts, which increase with age, make the thymic microenvironment unsuitable for the development of human prothymocytes.

A previous study in NOG mice suggested that, although human T cells have a normal phenotype, they are neither abletoproliferate nor to produce IL-2 in response to stimulation by anti-CD3/anti-CD28 antibodies [10]. The authors postulated that this abnormal function could be related to the involvement of murine MHC expressed on thymic epithelium in the selection of human T cells. In our experiments, after 22 weeks of engraftment, we did not observe such an anergy since human cells isolated from the lymph nodes responded satisfactorily to T cell stimulation. Interestingly, the responses were lower in the spleen than in the lymph nodes. This could be due to a lower proportion of T cells in this organ but also to anergic state of splenic T cells due to chronic response directed against HLA class II expressed by B cells as suggested by Watanabe *et al.* [10].

After intravenous inoculation of humanized NSG mice with HIV-1Bal and HIV-1JRCSF isolates, we observed high levels of viral replication and a rapid decline of CD4^+^ T cell counts. Further experiments will be needed to determine if our model can sustain the viral replication beyond 120 day despite dramatic CD4 lymphopenia. It has indeed been previously reported that Rag2^−/−^γc^−/−^ mice conditioned with TBI and transplanted with human CD34^+^ cells sustain high-titer infection persisting more than one year and associated with a slower decline of CD4 T cells after inoculation of an R5 strain [25], [26]. The reasons for different paces of CD4^+^ T cell depletion in both models are unknown.

We have observed an upregulation of various membrane markers associated with immune activation, senescence and exhaustion on the CD4^+^ and CD8^+^ T cells of our infected humanized mice. Similar findings have been previously reported with the humanized BLT-NSG mice [27], [28] and with Rag2^−/−^γc^−/−^ mice transplanted with HPCs [29] confirming that immune activation is a robust and cardinal feature of HIV infection in different settings. Interestingly, we were the first to describe in a murine model the dramatic increase of β_2_-microglobulin associated with the progression to human AIDS. Bacterial translocation of microbial products has been proposed to be a major cause for immune activation in HIV infection. After HIV infection of our HPCs transplanted humanized NSG mice, we indeed observed elevated plasma LPS levels. Hofer *et al*. recently reported similar findings in Rag2^−/−^γc^−/−^ mice [29]. Interestingly, our study showed plasma LPS below detection levels in non-infected humanized mice. This very low baseline of plasma LPS, crucial for further studies, might be related to our conditioning protocol since TBI conditioning is known to increase LPS levels in mice [30].

Modest but significant anti-HIV specific humoral immune responses could be detected after 9 weeks of infection in three mice. This could be due in part to an intrinsic defect of humoral responses in such murine models. Despite an increase of total IgG in the infected animals, these levels remained very low in comparison to what is observed in humans. This defect is likely to be due to the murine environment, which does not provide the critical growth factors necessary for proper B cells maturation and homeostasis [31]. This hypothesis is supported by our observation of a lower number of CD138^+^ immunoglobulin secreting plasma cells in the spleen. Choi *et al*. have reported that NSG mice engrafted after a dose of 30 mg/kg of busulfan have a better B cell development, which includes memory B cells than after conditioning with TBI or with a lower dose of busulfan [13]. Thus, it is possible that our conditioning protocol with busulfan at 50 mg/kg has probably contributed to the development of the modest anti-HIV-1 humoral responses seen in our humanized mice. Further improvement in B cell response could be achieved with treatment of the humanized NSG mice with B lymphocyte stimulator (BlyS/BAFF) to humanized NSG [31].

The high expression levels of perforin and granzyme B in CD8^+^ T cell suggests that these cells are functional and could play a role in control of HIV infection in humanized mice. The moderate number of IFN-γ secreting cells in our ELISPOT test further shows the presence of specific T cell responses against HIV antigens. However, T cells sorting and the use of lymph nodes rather than spleens as they have higher percentage of T cells, could allow the detection of the rare anti-HIV antigen specific T cells in humanized NSG mice.

Although our protocol is easy, efficient and reproducible, we fear that the need for HPCs obtained either fresh or cryopreserved in optimal conditions could impact its performance in some research settings. In previous publications of the Rag2−/−γc−/− mouse model (RAG-hu), human fetal liver-derived CD34^+^ cells were cultured for 1-day in cytokine media containing IL-3, IL-6 and SCF 2 and injected into neonatal mice after irradiation at 350 rads, intrahepatically [32]. These RAG humanized mice are productively infected by HIV-1. They produced continued long-term infection with X4-tropic or R5- tropic HIV-1 and have been evaluated for effective HIV-1 mucosal transmission [33]. Potential of similar protocol with busulfan conditioning and use of human cytokines pre-treated fetal liver derived CD34^+^ cells for transplantation could be assessed to humanize NSG mice were availability of fresh CD34^+^ cell is a limitation to achieve high engraftment levels.

Our new protocol of HPCs transplantation induces efficient engraftment in NSG mice. It not only improves the level and function of T cells and lymphoid organogenesis, but it also mimics HIV-1 replication, immune activation and pathogenesis mechanism as observed in human. However, generation of moderate anti-HIV specific IgG responses and CTL responses support the need for further improvement in CB-34^+^ cell transplanted humanized NSG mouse model.

Better anti-EBV CTL responses have been observed in newborn NSG-HLA-A2/HHD mice compared with NSG mice [34]. We have also started evaluating the same engraftment protocol described in this manuscript for future use in NSG-HLA-A2/HHD mice. Our preliminary results with NSG-HLA-A2/HHD mice suggest that our protocol of 50 mg/kg with two split doses of 25 mg/kg in 3–4 week old mice is well tolerated and able to produce high levels of engraftment in these mice as well. Thus, the protocol delineated in this article could be used with NSG-HLA-A2/HHD mice. This would be interesting since humanized NSG-HLA-A2/HHD mice have been shown to elicit strong T cell responses directed against HLA-A2-restricted dengue virus and EBV peptide [34], [35].

In summary, we have shown that high levels of human cell chimerism can be created in humanized NSG mice without the use of TBI. These mice can provide a valuable model to investigate HIV pathogenesis and immune activation mechanism and host immunity *in vivo* along with an opportunity to evaluate new therapeutic strategies.
